# Characterization of Proteins from Grain of Different Bread and Durum Wheat Genotypes

**DOI:** 10.3390/ijms12095878

**Published:** 2011-09-14

**Authors:** Slađana Žilić, Miroljub Barać, Mirjana Pešić, Dejan Dodig, Dragana Ignjatović-Micić

**Affiliations:** 1 Maize Research Institute, Zemun Polje, Slobodana Bajića 1, 11000 Belgrade-Zemun, Serbia; E-Mails: szilic@mrizp.rs (S.Ž.); ddodig@mrizp.rs (D.D.); idragana@mrizp.rs (D.I.-M.); 2 Faculty of Agriculture, University of Belgrade, Nemanjina 6, 11000 Belgrade-Zemun, Serbia; E-Mail: mpesic@agrif.bg.ac.rs

**Keywords:** bread wheat, durum wheat, protein fractions and subunits, tryptophan, wet gluten

## Abstract

The classical Osborne wheat protein fractions (albumins, globulins, gliadins, and glutenins), as well as several proteins from each of the four subunits of gliadin using SDS-PAGE analyses, were determined in the grain of five bread (*T. aestivum* L.) and five durum wheat (*T. durum* Desf.) genotypes. In addition, content of tryptophan and wet gluten were analyzed. Gliadins and glutenins comprise from 58.17% to 65.27% and 56.25% to 64.48% of total proteins and as such account for both quantity and quality of the bread and durum wheat grain proteins, respectively. The ratio of gliadin/total glutenin varied from 0.49 to 1.01 and 0.57 to 1.06 among the bread and durum genotypes, respectively. According to SDS-PAGE analysis, bread wheat genotypes had a higher concentration of α + β + γ-subunits of gliadin (on average 61.54% of extractable proteins) than durum wheat (on average 55.32% of extractable proteins). However, low concentration of ω-subunit was found in both bread (0.50% to 2.53% of extractable proteins) and durum (3.65% to 6.99% of extractable proteins) wheat genotypes. On average, durum wheat contained significantly higher amounts of tryptophan and wet gluten (0.163% dry weight (d.w.) and 26.96% d.w., respectively) than bread wheat (0.147% d.w. and 24.18% d.w., respectively).

## 1. Introduction

Wheat is one of the most important cereal crops worldwide in terms of production and utilization. It is a major source of energy, protein, and dietary fiber in human nutrition and animal feeding. It provides approximately one-fifth of the total calorific input of the World’s population [1]. Currently, about 95% of the wheat grown worldwide is hexaploid bread wheat, with most of the remaining 5% being tetraploid durum wheat.

The ability of wheat flour to be processed into different foods is largely determined by the proteins. Mature wheat grains contain 8% to 20% proteins. Wheat proteins show high complexity and different interactions with each other, thus making them difficult to characterize. Usually, they are classified according to their solubility. Following the sequential Osborne extraction procedure, albumins, globulins, gliadins and glutenins are isolated. An alternative classification to that described above has been proposed based on composition and structure rather than solubility [2].

Albumins and globulins of wheat endosperm represent 20% to 25% of total grain proteins [3,4]. Nutritionally, the albumins and globulins (non-glutens) have a very good amino acid balance. Many of these proteins are enzymes involved in metabolic activity. However, several other proteins have unknown functions and are not well characterized. Some proteins, particularly those belonging to a family of trypsin and α-amylase inhibitors, are also implicated in plant defense [5], but the role of α-amylase and trypsin inhibitors as wheat allergens in baker’s asthma has been demonstrated [6]. Most of the physiologically active proteins also influence the processing and rheological properties of wheat flour. In recent years, the benefits of the use of amylases, xylanases, lipoxygenase, pentosanase, glucoseoxidase, has stimulated further interest in the bread-making industry [7,8].

Wheat is unique among the edible grains because wheat flour has the protein complex called “gluten” that can be formed into dough with the rheological properties required for the production of leavened bread [9]. The rheological properties of gluten are needed not only for bread production, but also in the wider range of foods that can only be made from wheat, viz., noodles, pasta, pocket breads, pastries, cookies, and other products [10]. The gluten proteins consist of monomeric gliadins and polymeric glutenins. Glutenins and gliadins are recognized as the major wheat storage proteins, constituting about 75–85% of the total grain proteins with a ratio of about 1:1 in common or bread wheat [3,11] and they tend to be rich in asparagine, glutamine, arginine or proline [12] but very low in nutritionally important amino acids lysine, tryptophan and methionine [13].

The gliadins constitute from 30 to 40% of total flour proteins and are polymorphic mixture of proteins soluble in 70% alcohol, and can be separated into α-, β-, γ-, and ω-gliadins with a molecular weight range of 30 to 80 kDa as determined by SDS-PAGE. The molecular weights of ω-gliadins are between 46 and 74 kDa, and the α-, β- and γ-gliadins have lower Mw, ranging from 30 to 45 kDa by SDS-PAGE and amino acid sequencing [14]. The latter approach has shown that the α- and β-gliadins are closely related and thereby they are often referred to as α-type gliadins. α-Gliadins are thought to be responsible for gluten intolerance [15] while γ-gliadins and glutenins are much less [16].

Glutenin polymers are made up of single polypeptides linked through intermolecular disulfide bonds that account for about 45% of the total proteins in the grain endosperm. Glutenins can be broadly classified into two groups, the high molecular weight (HMW) and the low molecular weight (LMW) subunits, with molecular weight (Mw) range of 100 to 140 kDa and 30 to 55 kDa, respectively, according to mobility on SDS-PAGE [17]. They link together and form heterogeneous mixtures of polymers by disulfide bonded linkages of polypeptides. The glutenin proteins, therefore, are among the largest protein molecules in nature with molecular weights up into tens of millions [18]. Differences in glutenin subunits size, polarity, and number of cysteine residues influence the ability to form disulfide bonds necessary for building up the glutenin polymer structure. This variation in glutenin subunits is a critical factor in determining bread dough end-product quality, particularly through its influence on polymer size distribution [19]. The LMW subunits most closely resemble γ-gliadins in sequence [20] and comprise about 20% to 30% of the total proteins while the HMW subunits account for about 5 to 10% of the total proteins [21].

The goals of this study are as follows: (i) to evaluate the magnitude of the classical Osborne wheat protein fractions (albumins, globulins, gliadins, and glutenins) across the grain of five bread and five durum wheat genotypes; (ii) to determine different protein components of the four subunits of wheat gliadin using SDS-PAGE analyses; (iii) to determine the content of tryptophan as an aromatic and essential amino acid and wet gluten as a quality parameter of wheat flour. A more detailed knowledge of the variability of proteins and protein fractions accumulation among new varieties, could facilitate ongoing efforts to improve both quantity and quality of wheat proteins and could influence the selection of better raw materials for the flour and bread-making industry. Furthermore, to be able to use whole wheat flour in production of functional food, rich in health-beneficial components, the study of the whole grain proteins content, their structure and quality are important.

## 2. Experimental Section

### 2.1. Wheat Samples

The experimental material consisted of four bread (*Triticum aestivum* L.) and four durum (*Triticum durum* Desf.) wheat genotypes (breeding lines and cultivars) recently developed at the Maize Research Institute Zemun Polje (MRIZP), Serbia. The genotypes were chosen on the basis of their differences in agronomic traits such as yield and its components. In addition, one bread (recently wide spread in Serbia) and one durum (good pasta quality) foreign cultivar was used for comparison. Their names, pedigrees, origin and growth type are given in Table 1. Grain samples of bread and durum wheat were collected from plants grown in a field-trial at the MRIZP in 2009–2010 growing season. The experiment was laid out in the randomized complete block design (RCBD) with two replications. Each plot consisted of eight 5 m rows at 12.5 cm spacing (machine sowing). Standard agronomic practices were used to provide adequate nutrition and to keep the plots free of diseases.

For the analysis of both wheat species, the wholemeal (particle size < 500 μm) was obtained by grounding wheat grains on a Cyclotec 1093 lab mill (FOSS Tecator, Sweden).

### 2.2 Chemical Analysis

#### 2.2.1. Osborne Fractionation Method

##### Albumin-Globulin extraction

Defatted wheat flour (0.5 g) was sequentially extracted by the Osborne procedure described by [22] (Lookhart and Bean, 1995) with modifications. The flour was extracted with an aqueous solution of 0.5 M NaCl (10 mL). Extraction was done by repeated stirring three times for 30 min at 4 °C, followed by centrifugation at 20,000 g for 15 min. All supernatants (albumin + globulin (Alb) (Glob) extracts) were transferred to the volumetric flask and 0.5 M NaCl was added to 50 mL. The centrifugate was vortexed with deionized water (10 mL) for 1 min, than set for 5 min, centrifugated, and the supernatants discarded. This additional wash was made with water to reduce the effect of the salt in the pellet for the extraction of gliadin in the following steps.

##### Gliadin extraction

The water-washed pellet from globulin was extracted with 70% aqueous ethanol (10 mL) for 30 min at 4 °C. The ethanol solution mixture was centrifuged for 15 min at 20,000 g. Extraction was done three times, the supernatants (gliadin (Gli) extracts) were transferred to the volumetric flask and 70% ethanol was added to 50 mL.

##### Soluble glutenin extraction

Glutenins (Glu) were extracted from the gliadin pellet in three steps in a similar way with 7 mL of 50% 1-propanol + 1% dithiothreitol (DTT). Yield of Glu-1, Glu-2 and Glu-3 extracts were transferred to the volumetric flask and extraction solution was added to 25 mL. Glutenin extracts are built up from high molecular weight (HMW) and low molecular weight (LMW) glutenin subunits, but the bulk of the soluble glutenins consists of LMW glutenin subunits [23].

##### Insoluble glutenin

Content of insoluble glutenin was calculated as a difference between content of total protein and sum of albumin + globulin, gliadin and soluble glutenin.

Protein content was calculated, in each fraction, from the nitrogen content determined by micro Kjeldahl method, using 5.7 as the conversion factor. The results are given as percentage of dry weight (d.w.), as well as percentage of total protein (protein solubility index-PSI).

#### 2.2.2. SDS-PAGE Gel Electrophoresis

Extractable protein composition of the defatted samples was detected by the sodium dodecyl sulfate-polyacrilamide gel electrophoresis (SDS-PAGE) performed according to Fling and Gregerson [24], on 12.5% separating gels and 5% stacking gels in vertical electrophoretic unit (LKB, Sweden). Gliadins was extracted by the Osborne procedure described by Lookhart and Bean [22]. Prior to the electrophoresis, extractable proteins have been diluted in the ratio 1:2 (v/v) with the sample buffer (0.055 M Tris-HCl, pH 6.8, 2% (w/v) sodium dodecyl sulfate (SDS), 20% (v/v) glycerol, 4.3% (v/v) β-mercaptoethanol, 0.0025% (w/v) bromophenol blue), heated at 90 °C for 5 min and cooled at the room temperature. Fifty microliters of gliadin fraction were loaded per well. Gels were run at 50 mA for five hours, fixed and stained with 0.23% (w/v) Coomassie Blue R-250 dissolved in 3.9% (w/v) trichloroacetic acid (TCA), 6% (v/v) acetic acid and 17% (v/v) methanol for 45 min. Destaining was performed with 8% acetic acid and 18% (v/v) ethanol. Molecular weights of the polypeptides were estimated by using low molecular weight standards (Amercham Biosciences, Sweden): phosphorylase B (94.0 kDa), bovine albumin (66.0 kDa), ovalbumin (45.0 kDa), carbonic anhydrase (30.0 kDa), soybean trypsin inhibitor (20.1 kDa), and α-laktalbumin (14.4 kDa). The protein bands on the destained gel were quantitated using SigmaGel sotware version 1.1 (Jandal, San Rafael, CA). The concentration of wheat proteins and their ration were calculated from the sum of the total area of their subunits and expressed as percentage of total extractable proteins. To investigate varietals effect, electrophoresis was performed in triplicate. Namely, three aliquots of the same sample were analyzed at the same time. Two gels were run simultaneously in the same electrophoretic cell. Also, three replications of extraction procedure were performed.

#### 2.2.3. Wet Gluten Content

Wet gluten content (%) is determined by washing the dough obtained from wheat flour (10 g), with 2% NaCl solution, followed by water in certain conditions, to remove the starch and other soluble compounds of the sample [25].

#### 2.2.4. Tryptophan Content

Tryptophan content was determined according to Nurit *et al.* [26] from defatted wheat flour. Shortly, flour hydrolysate (obtained by overnight digestion with papain solution at 65 °C) was added to 3 mL reagent containing Fe^3+^ (1 g FeCl_3_ dissolved in 50 mL 3.5 M H_2_SO_4_), 15 M H_2_SO_4_ and 0.1 M glyoxilic acid. After incubation at 65 °C for 30 min, absorption was read at 560 nm. Tryptophan content was calculated using a standard (calibration) curve, developed with known amounts of tryptophan, ranging from 0 to 30 μg mL^−1^. The standard chemical methods were applied to determine the content of total proteins. Besides tryptophan content quality index (QI), defined as tryptophan to protein ratio in the sample, was also calculated.

#### 2.2.5. Statistical Analyses

All chemical analyses were performed in three replicates per plot and the results were statistically analysed. Significant differences between genotype means were determined by the Fisher’s least significant differences (LSD) test at, after the analysis of variance (ANOVA) for trials set up according to the RCB design (MSTAT-C). A *t*-test was performed to test the significance of differences between the species means. Differences with *P* < 0.05 were considered significant in both tests. The coefficient of variation (CV) was determined for each trait.

## 3. Results and Discussion

### 3.1. Results

Data in Table 2 indicate that the content of total proteins was significantly higher in durum (on average 11.81% d.w. of defatted flour) than bread (on average 11.08% d.w. of defatted flour) wheat genotypes. However, higher variation for this trait was observed among bread than durum wheat genotypes (13.54% *vs.* 5.71%).

No significant differences in the mean of AG (albumin + globulin), as well as gliadin content were observed between bread and durum wheat. The average values of bread and durum wheat samples for the PSI of AG fraction were 38.45% and 38.63%, respectively. The gliadin content of bread and durum wheat samples was lower than AG content and ranged from 21.44% (ZP *Zemunska rosa*) to 29.84% (ZP Zlatna), and 21.98% (ZP 34/I) to 28.90% (ZP DSP/01) of total proteins, respectively. A slightly higher variation for gliadin was found in bread (14.88%) than in durum (10.17%) wheat genotypes (Table 2).

The protein fraction with the lowest PSI was soluble glutenin, in all the analyzed genotypes (Table 2). The content of soluble glutenins ranged from 9.49% to 14.91% of total proteins and from 7.24% to 11.69% of total proteins in wholemeal of bread and durum wheat genotypes, respectively. The significant difference (*P* < 0.05) was observed between means of bread and durum wheat (11.58% and 9.54% of total proteins, respectively). Also, there was significant difference in the mean content of insoluble glutenin between bread and durum wheat (Table 2). On average, content of insoluble glutenin was 26.76% and 24.59% of total proteins in durum and bread wheat, respectively. Relatively high variations for content of soluble and insoluble glutenin of total proteins were found within both bread (17.28% and 29.92%, respectively) and durum (15.97% and 18.50%, respectively) wheat genotypes. According to our study, the gliadins and total glutenins constitute from 58.17% to 65.26% and 56.25% to 64.48% of total grain proteins, with a ratio from 0.49 to 1.01 and 0.57 to 1.06 in bread and durum wheat genotypes, respectively.

**Figure 1:**
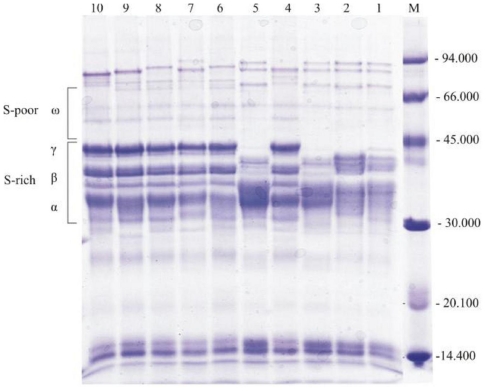
SDS-PAGE patterns gliadins from bread and durum wheat genotypes. ω, γ, β and α indicate subunits of gliadin. 1–5 bread wheat: 1—ZP 87/I, 2—Apache, 3—ZP *Zemunska rosa*, 4—ZP 224, 5—ZP Zlatna; 6–10 durum wheat: 6—ZP 34/I, 7—ZP 10/I, 8—ZP DSP/01, 9—Varano, 10—ZP 7858, M—Molecular weight standards.

Gliadins and glutenins are recognized as the major wheat storage proteins. To identify variants of storage gliadin subunits in bread and durum genotypes, protein extracts were analyzed by SDS-PAGE and electrophoretic patterns of gliadin extractable proteins are shown in Figure 1. The gliadin polypeptide composition of bread and durum genotypes is shown in Table 3.

The protein bands were different among all the wheat genotypes. The bread and durum grain polypeptides with molecular weight between 31.4 and 43.9 kDa belong of α-, β- and γ-subunits of gliadin. These S-poor subunits were consisted of three to six polypeptides depend on genotypes. However, the resolution of proteins’ region between 34.8 and 37.6 kDa was not clear enough to detect several components separately. The group of polypeptides with a molecular weight of about 55.7 to 73.6 kDa is S-poor subunits of gliadin or ω-gliadins. According to our results, ω-gliadin subunits were consisted of one to three polypeptides with a molecular weight of 55.7, 62.4 and 73.6 kDa (Figure 1).

Significant differences between bread and durum genotypes for S-rich subunits, as well as S-poor subunits concentration were determined by the densitometric analysis (Table 4). In both species, S-rich subunits were the most abundant gliadin subunits. The S-rich subunits concentration of bread wheat genotypes ranged from 54.75% (ZP *Zemunska rosa*) to 67.09% (ZP 224), with an average value of 61.54% of total extractable proteins. Among the tested durum wheat genotypes, the highest S-rich subunits concentration of 60.78% of total extractable proteins was detected in ZP 34/I, whereas the lowest concentration of 52.56% of total extractable proteins was detected in ZP 10/I. The average value of durum wheat genotypes for the S-rich subunits concentration was 55.32% of total extractable proteins, which was for about 10% lower than that of bread wheat. The mean S-rich subunits concentration did not vary much among bread and durum wheat genotypes (7.24% and 5.23%, respectively). However, considerable variation for the S-poor subunits concentration was found among bread and durum wheat genotypes (57.29% and 39.96%). In average, durum wheat grain had significantly higher concentration of S-poor subunits (5.73% of total extractable proteins) than bread wheat grain (1.28% of total extractable proteins). The sum of gliadins’ S-poor and S-rich subunits ranged from 55.58% to 69.62% and 58.65% to 64.43% of total extractable proteins in bread and durum wheat genotypes, respectively, and no significant differences were observed between means.

Besides major protein subunits, polypeptides with molecular weight of 111.3 kDa, 101.2 kDa, 93.5 kDa, 90.5 kDa, 87.7 kDa, 85.7 kDa, 82.3 kDa, 80.3 kDa, 77.2 kDa, 29.7 kDa, 29.1 kDa, 26.6 kDa, 20.6 kDa, 16.2 kDa, 15.0 kDa, 14.0 kDa, 11.4 kDa were detected by SDS-PAGE. These proteins were not presented in all varieties (Figure 1, Table 3).

Results of the tryptophan in bread and durum wheat genotypes determined by colorimetric method and levels of statistical significance obtained from analysis of variance, are summarized in Table 5. In average, durum wheat grain had a significantly higher level of tryptophan (0.163% d.w.) than bread wheat grain (0.147% d.w.). However, there was a similar QI between bread and durum wheat, although significant differences were observed among genotypes within each species.

**Figure 2:**
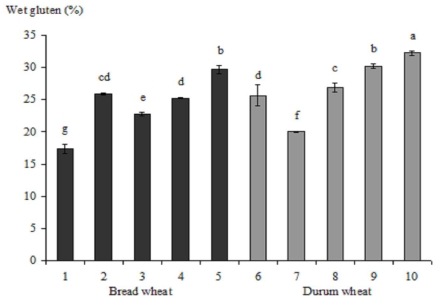
The content of wet gluten in different wheat varieties. 1–5 bread wheat: 1—ZP 87/I, 2—Apache, 3—ZP *Zemunska rosa*, 4—ZP 224, 5—ZP Zlatna; 6–10 durum wheat: 6—ZP 34/I, 7—10/I, 8—ZP DSP/01, 9—Varano, 10—ZP 7858. Bars with different letters are statistically significantly different (*P* < 0.05).

The content of wet gluten of bread and durum wheat wholemeal is shown in Figure 2. Durum wheat contained significantly higher amount of wet gluten than bread wheat (26.96% *vs.* 24.18%). Wet gluten ranged from 17.35% (ZP 87/I) to 29.65% (ZP Zlatna) and 20.00% (ZP 10/I) to 32.20% (ZP 7858) in bread and durum genotypes, respectively.

### 3.2. Discussion

The major emphasis in wheat has been on high protein wheat for nutritional enhancement and improved processing performance. Vogel *et al.* [27] reported that protein content of 12,600 wheat lines from the USDA World Wheat Collection ranged from about 7% to 22% d.w., with the genetic component accounting for about a third of this (*i.e.*, about 5%). The greater part of the variation was due to non-genetic factors and this strong environmental impact has made breeding for high protein difficult [13]. Due to the small number of analyzed genotypes in our study, the total protein contents varied in the significantly narrower range, *i.e.*, 9.26% to 12.64%. Because grains were collected from plants grown under equal conditions in a field-trial at the same location during the same growing season, the influence of environmental factors could be ignored. However, it should be noted that rainfalls from anthesis to maturity in the season of trial (2009–2010) probably caused overall reduction in protein content. The mean total proteins did not vary much among durum wheat genotypes (5.71%), but relatively a high variation was found among bread wheat genotypes (13.54%).

The ability of wheat flour to be processed into different foods is largely determined by the gliadins and glutenins [28] which constitute up to 63–90% of the total grain proteins [29]. Because of their unique viscoelastic properties, gliadins and glutenins are responsible for the bread-making quality of wheat flour [18]. In the present study, gliadins and glutenins in grain of bread wheat ranged from 58.17% (Apache) to 65.26% (ZP *Zemunska rosa*) of total proteins. According to Stehno *et al.* [30] and Abdelrahman *et al*. [31], gliadins and glutenins constitute from 69.12% to 77.71% and 65.83% to 66.36% of the total grain proteins in cultivars grown in Czech Republic and Sudan, respectively. Among durum wheat genotypes, content of gliadins and glutenins was the highest in grain of ZP 10/I (64.48% of total proteins). Also, this genotype had the highest content of soluble glutenin (11.69% of total proteins) which mainly consist of low molecular weight proteins. According to Kovacs *et al.* [32] the pasta cooking quality and gluten strength were initially related to the beneficial effects of LMW-2 glutenin. The high molecular glutenin subunits of wheat grain are of immense importance in determining the quality and the end use properties of the dough [33]. Two features of HMW subunits structure may be relevant to their role in glutenin elastomers: the number and distribution of disulphide bonds and the properties and interactions of the repetitive domains [34]. Among analyzed genotypes, the highest content of insoluble glutenin, which mainly consist of high molecular weight polypeptides, had bread wheat cultivar ZP *Zemunska rosa* (34.33% of total proteins). However, this genotype had the lowest gliadin/glutenin ratio (0.49). ZP *Zemunska rosa* is generally considered as wheat of not excellent, but sufficient, bread making quality. From measurements on glutens reconstituted at various glutenin/gliadin ratios, Janssen *et al.* [35] found that, at constant protein content, the main factor determining the rheological behavior of hydrated gluten is the glutenin to gliadin ratio. Generally, it is believed that gliadin controls the viscosity of the dough and glutenin controls the elastic or strength properties [36]. The precise balance between viscosity (extensibility) and elasticity (dough strength) is important for bread-making. The gliadin/glutenin ratio range (0.49 to 1.01) obtained for bread wheat genotypes grown in Serbia were similar with that of 0.59 to 0.84 reported by Stehno *et al.* [30] for ten bread cultivars grown in Czech Republic. The highest content of gliadin (29.84% and 29.27% of total proteins), as well as gliadin/glutenin ratio (0.97 and 1.01), were in grain of ZP Zlatna and Apache, respectively. ZP Zlatna is registered in Serbia and classed as high bread-making quality (class A). Also, these genotypes had the lowest content of insoluble glutenin and the highest content of AG protein fractions. Although the albumin and globulin fractions are not known to play a direct role in bread-making, as gluten proteins, they may be necessary for normal baking properties [37]. However, in comparison with the gluten proteins, albumins and globulins have a better spectrum of essential amino acids (lysine, arginine, aspartic acid, threonine and tryptophan).

According to alternative classifications, wheat gluten can be separated into three large groups: sulfur-rich (Mw of ~50 kDa; α-, β-, γ-gliadins and B- and C-LMW glutenins), sulfur-poor (Mw ~50 kDa; ω-gliadins and D-LMW glutenins) and high molecular weight (Mw ~100 kDa; HMW glutenins) proteins. In our study, significant differences between bread and durum wheat for S-poor subunits, as well as S-rich gliadin subunits concentration were determined. Although the distribution of total gliadins among the different types is strongly dependent on wheat genotypes and growing conditions, it can be generalized that α/β- and γ-gliadins are major components, whereas the ω-gliadins occur in much lower proportions [38]. In our work, both species had considerable higher concentration of S-rich gliadin subunits. The Mw of bread and durum gliadins ranged from 31.4 to 73.6 kDa. This range was in agreement with values (34 to 75 kDa) reported by Abdel-Aal *et al.* [11]. It was obvious that there were differences in the number of ω-gliadin bands between bread and durum wheat. The bread wheat was characterized by the presence of weak intensity ω-gliadin bands. However, the bread wheat genotypes had a distinct strong band in α/β-region with molecular weight of about 37.6 to 34.8 kDa. This finding agrees with Federmann *et al.* [39]. There was strong-staining band or polypeptide chain with molecular weight of about 42 to 44 kDa that appeared in all durum wheat genotypes. This polypeptide chain was in the γ-gliadin region and was absent in bread wheat genotypes, except in the grain of genotype ZP 224. This is in agreement with Abdel-Aal *et al.* [11] who detected that this band was absent in common wheat. Therefore, this γ-gliadin band might be used to differentiate the durum from bread wheat. The Mw ranged from 77.2 to 111.3 kDa, indicating that bread and durum gliadin extracts contained HMW components which could be polypeptide chains of glutenin. These polypeptides regions consisted of 1–3 sharp, thin bands with MW’s of 94–111 kDa. Chakraborty and Khan [40] have shown by SDS-PAGE that some glutenins are extracted with 70% aqueous ethanol.

Most ω-gliadins lack cysteine, so that there is no possibility of disulphide crosslinks. These proteins consist almost entirely of repetitive sequences rich in glutamine and proline. However, α/β-gliadins contain six, and γ-gliadins eight, cysteines located in the C-terminal domain and they form three and four homologous intrachain crosslinks, respectively [41]. It is known that the ratio of α/β- and γ-gliadins to ω-gliadin influence the sulfur amino acid content, quality of wheat grain proteins and structure and functionality of gluten. In this study, the ratio of S-poor/S-rich gliadin subunits varied from 0.008 to 0.038 and 0.060 to 0.130 among the bread and durum genotypes, respectively. In durum wheat a highly significant correlation has been detected between specific durum wheat γ-gliadin and gluten strength. γ-Gliadins 45 and 42 are useful markers for good and poor pasta quality, respectively and this is due to the genetic linkage with low molecular weight glutenin subunits [42]. On the other hand, β-gliadin subunits may be associated with elevated loaf volumes, and could be the target for indirect selection for breeding programs improving durum wheat for bread-making quality. Generally, a high content of gliadins in the grain gives a poor nutritional quality of the flour, because the gliadins are a very poor source of lysine, tryptophan, and sulfur containing amino acids [29]. In addition to sulfur amino acid, reliable information concerning the tryptophan content of cereals is essential, especially when cereals are a major source of proteins. Tryptophan plays a role as a precursor of the neurotransmitter serotonin and the epiphyseal hormone melatonin [43]. According to our study, tryptophan constitutes from 1.171% to 1.621% and 1.245% to 1.496% of total grain proteins in bread and durum wheat genotype, respectively. Obtained contents for tryptophan in analyzed wheat genotypes were lower than the contents reported by Gafurova *et al.* [44] (1.8% to 2.0% of total grain proteins). Nevertheless, our results are similar to those reported by Comai *et al.* [45] for the tryptophan content of different wheat varieties purchased from the Italian market (on average 1.160% of total proteins).

## 4. Conclusions

The quality of wheat protein is genetically determined and varies between wheat cultivars and species. There were significant differences in the mean soluble and insoluble glutenin between bread and durum wheat. The genotypic variation in the contribution of glutenin to bread-making quality is due to variation in the number of specific HMW subunits. The results of SDS-PAGE showed that concentration of α/β-, γ-gliadins and ω-gliadin was statistically significant among bread and durum wheat. The bread wheat had weak intensity of ω-gliadin band and strong band in α/β-region. Also, the polypeptide chain in the γ-gliadin region was absent in bread wheat, except in the grain of genotype ZP 224. It appears that even among small samples of bread and durum genotypes considerable differences in amount of protein fractions can be found that could be manipulated in the future for a desired level of the protein components. However, more research is needed to evaluate the effects of different sites and years on the wheat protein characteristics in order to quantify the environmental factors affecting these characteristics. Also, the breeders would need to evaluate their own materials in their own geographic regions before definitive decisions could be made in their breeding programs.

## Figures and Tables

**Table 1:** Name, pedigree, growth type and origin of bread and durum genotypes; country code from the UN website.

Genotypes	Parents (Origin)	Country	Growth Type
Bread wheat			
ZP 87/I	L-99 (SRB) × Pobeda (SRB)	SRB	winter
ZP *Zemunska rosa*	Skopljanka (MKD) × Proteinka (SRB)	SRB	winter
ZP 224	L-4 (SRB) × Dulus/Metso (CIMMYT)	SRB	facultative
ZP Zlatna	Jasenica (SRB) × Rodna (SRB)	SRB	winter
Apache		FRA	winter

Durum wheat			
ZP 34/I	SOD 55 (SVK) × Korifla (ICARDA)	SRB	facultative
ZP 10/I	Windur (DEU) × Rodur (ROU)	SRB	winter
ZP DSP/01	Windur (DEU) × SOD 64 (SVK)	SRB	winter
ZP 7858	Mina (MKD) × Mexicali 75 (CIMMYT)	SRB	facultative
Varano		ITA	facultative

ICARDA = International Center for Agricultural Research in the Dry Areas (SYR); CIMMYT = International Maize and Wheat Improvement Centre (MEX).

**Table 2:** The content of protein fractions in grains of different wheat varieties.

Varieties	Protein	Albumins + Globulins		Gliadins		Soluble glutenins		Insoluble glutenins		Sum Gli + Glu	Ratio Gli/Glu

	(1)	(1)	(2)	(1)	(2)	(1)	(2)	(1)	(2)	(2)	
Bread wheat											
ZP 87/I	9.51 ^d^	3.51 ^e^	36.93 ^c^	2.31 ^c^	24.31 ^b^	1.16 ^d^	12.20 ^b^	2.53 ^c^	26.56 ^b^	63.07 ^b^	0.62 ^b^
Apache	9.26 ^d^	3.87 ^d^	41.79 ^a^	2.71 ^b^	29.27 ^a^	1.38 ^a^	14.91 ^a^	1.29 ^d^	13.99 ^d^	58.17 ^d^	1.01 ^a^
ZP *Zemunska rosa*	12.64 ^a^	4.39 ^c^	34.73 ^d^	2.71 ^b^	21.44 ^c^	1.20 ^c,d^	9.49 ^d^	4.34 ^a^	34.33 ^a^	65.26 ^a^	0.49 ^c^
ZP 224	11.76 ^c^	4.62 ^b^	39.28 ^b^	2.59 ^b^	21.93 ^c^	1.30 ^a,b^	11.01 ^c^	3.25 ^b^	27.63 ^b^	60.57 ^c^	0.57 ^b,c^
ZP Zlatna	12.22 ^b^	4.83 ^a^	39.52 ^b^	3.63 ^a^	29.84 ^a^	1.26 ^b,c^	10.31 ^c,d^	2.50 ^c^	20.45 ^c^	60.66 ^c^	0.97 ^a^
*F*-test	***	***	***	***	***	*	***	***	***	***	***
CV (%)	13.54	12.11	6.67	16.84	14.88	6.73	17.28	38.14	29.92	13.85	31.81
Durum wheat											
ZP 34/I	12.15 ^a^	4.79 ^ab^	39.44 ^b^	2.67 ^c^	21.98 ^c^	0.88 ^e^	7.24 ^d^	3.81 ^a^	31.33 ^a^	60.55 ^c^	0.57 ^c^
ZP 10/I	11.12 ^b^	3.95 ^d^	35.52 ^d^	2.87 ^b^	25.81 ^b^	1.30 ^a^	11.69 ^a^	3.00 ^d^	26.98 ^c^	64.48 ^a^	0.67 ^b^
ZP DSP/01	11.04 ^b^	4.83 ^a^	43.75 ^a^	3.19 ^a^	28.90 ^a^	1.06 ^d^	9.55 ^bc^	1.96 ^e^	17.80 ^d^	56.25 ^d^	1.06 ^a^
Varano	12.36 ^a^	4.59 ^c^	37.15 ^c^	3.15 ^a^	25.76 ^b^	1.12 ^c^	9.16 ^c^	3.50 ^c^	28.29 ^c^	63.21 ^a,b^	0.68 ^b^
ZP 7858	12.40 ^a^	4.62 ^b,c^	37.28 ^c^	2.87 ^b^	23.16 ^c^	1.26 ^b^	10.04 ^b^	3.64 ^b^	29.41 ^b^	62.61 ^b^	0.58 ^c^
*F*-test	*	***	***	***	***	***	***	***	***	***	***
CV (%)	5.71	7.43	7.86	7.07	10.17	14.22	15.97	21.94	18.50	15.35	27.69

Mean (bread wheat)	11.08 ^b^	4.24 ^a^	38.45 ^a^	2.79 ^a^	25.36 ^a^	1.26 ^a^	11.58 ^a^	2.78 ^b^	24.59 ^b^	61.53 ^a^	0.73 ^a^
Mean (durum wheat)	11.81 ^a^	4.56 ^a^	38.63 ^a^	2.95 ^a^	25.12 ^a^	1.12 ^b^	9.54 ^b^	3.16 ^a^	26.76 ^a^	61.42 ^a^	0.69 ^a^

Mean of genotypes and species followed by the same letter within same column are not significantly different (*P* < 0.05);

* = significant at *P* < 0.05;

*** Significant at *P* < 0.001; CV, coefficient of variation; (1) % of dry weight; (2) % of total proteins.

**Table 3:** The polypeptide composition of gliadin fraction in grains of different wheat genotypes identified by SDS-PAGE (% of total extractable proteins).

Polypeptides Mw (kDa)	Bread wheat					Durum wheat					LSD_0.05_	CV (%)
	ZP 87/I	Apache	ZP *Zemunska rosa*	ZP 224	ZP Zlatna	ZP 34/I	ZP 10/I	ZP DSP/01	Varano	ZP 7858		
111.3	n.d.	n.d.	n.d.	n.d.	n.d.	n.d.	0.81 ^b^	1.23 ^a^	0.99 ^b^	0.81 ^b^	0.056	57.26
101.2	n.d.	n.d.	n.d.	n.d.	n.d.	n.d.	n.d.	n.d.	0.79 ^b^	1.51 ^a^	0.085	88.56
93.55	0.76 ^c^	1.38 ^b^	0.54 ^e^	n.d.	0.67 ^d^	n.d.	1.51 ^a^	n.d.	n.d.	n.d.	0.072	66.25
90.5	n.d.	n.d.	n.d.	n.d.	n.d.	1.23 ^b^	n.d.	2.81 ^a^	n.d.	n.d.	0.148	93.71
87.7	0.97 ^e^	1.08 ^d^	1.15 ^d^	2.06 ^c^	1.04 ^e^	n.d.	2.91 ^b^	1.07 ^d^	3.69 ^a^	n.d.	0.101	71.06
85.7	n.d.	n.d.	n.d.	n.d.	n.d.	n.d.	n.d.	n.d.	n.d.	4.14 ^a^	-	-
82.3	n.d.	n.d.	n.d.	0.64 ^b^	n.d.	n.d.	n.d.	n.d.	n.d.	1.40 ^a^	0.101	92.73
80.3	n.d.	n.d.	n.d.	n.d.	n.d.	0.57 ^d^	1.52 ^b,c^	1.40 ^c^	1.71 ^a^	1.39 ^c^	0.138	58.17
77.2	1.26 ^c^	1.43 ^b^	1.15 ^d^	0.59 ^e^	1.32 ^c^	0.36 ^f^	n.d.	n.d.	n.d.	1.64 ^a^	0.072	58.27
73.6	n.d.	n.d.	n.d.	n.d.	0.14 ^c^	0.56 ^b^	2.09 ^a^	2.05 ^a^	2.17 ^a^	n.d.	0.202	85.33
62.4	0.60 ^g^	0.50^g^	0.83^f^	1.28 ^e^	1.81 ^c^	1.77 ^c^	2.46 ^a^	2.29 ^b^	1.51 ^d^	2.24 ^b^	0.124	45.92
55.7	n.d.	n.d.	n.d.	1.25 ^e^	n.d.	1.32 ^e^	2.30 ^c^	2.65 ^b^	2.02 ^d^	3.24 ^a^	0.101	56.88
43.9	0.51	n.d.	n.d.	16.27 ^a^	n.d.	13.70 ^b^	9.95	12.92 ^c^	13.21 ^b,c^	13.64 ^b^	0.613	60.43
42.7	n.d.	n.d.	n.d.	0.76 ^c^	n.d.	3.17 ^a^	3.25 ^a^	1.38 ^b^	1.43 ^b^	1.50 ^b^	0.160	69.65
40.2	5.89 ^b^	17.19 ^a^	5.17 ^b^	n.d.	5.37 ^b^	n.d.	n.d.	n.d.	n.d.	n.d.	0.529	88.67
39.5	10.87 ^d^	7.31 ^e^	2.36 ^g^	12.37 ^b^	3.84 ^f^	13.44 ^a^	11.63 ^c^	10.70 ^d^	10.76 ^d^	11.41 ^c^	0.515	38.75
37.6–34.8	41.88 ^c^	32.43 ^d^	47.22 ^a^	31.81 ^d^	46.12 ^b^	24.03 ^e^	20.34 ^h^	22.59 ^f^	21.97 ^g^	23.15 ^e,f^	0.896	32.73
31.4	4.26 ^f^	6.43 ^b^	n.d.	5.88 ^c^	3.78 ^g^	6.44 ^b^	7.39 ^a^	5.03	5.58 ^d^	5.28 ^e^	0.202	40.07
29.7	0.70 ^f^	0.87 ^f^	4.03 ^a^	1.51 ^e^	1.86 ^d^	2.43 ^b,c^	2.37 ^c^	2.71 ^b^	n.d.	2.78 ^b^	0.263	59.71
29.1	n.d.	n.d.	n.d.	n.d.	n.d.	n.d.	n.d.	n.d.	1.77 ^a^	n.d.	-	-
26.6	n.d.	0.75 ^g^	1.22 ^f^	3.08 ^d^	0.81 ^g^	3.74 ^c^	2.71 ^e^	4.68 ^b^	4.87 ^b^	5.71 ^a^	0.252	70.45
20.6	n.d.	n.d.	n.d.	n.d.	0.14 ^f^	0.58 ^e^	2.65 ^b^	2.45 ^c^	3.13 ^a^	1.92 ^d^	0.189	80.08
16.2	0.48 ^g^	12.32 ^c^	13.45 ^b^	6.23 ^f^	14.14 ^a^	6.39 ^f^	5.89 ^f^	8.17 ^d^	7.02 ^e^	6.60 ^e,f^	0.529	50.19
15.0	13.39 ^b^	11.97 ^c^	15.09 ^a^	10.45 ^d^	11.28	12.21 ^c^	10.28 ^d^	8.85 ^f^	9.76 ^e^	7.82 ^g^	0.515	20.18
14.0	12.37 ^a^	4.67 ^c^	5.39 ^b^	2.85 ^f^	5.39 ^b^	4.69 ^c^	5.35 ^b^	3.69 ^d^	3.95 ^d^	3.14 ^e^	0.409	51.10
11.4	6.04 ^a^	1.67 ^g^	2.37 ^f^	2.95 ^e^	2.26 ^f^	3.33 ^d^	4.56 ^b^	3.32 ^d^	3.65 ^c^	0.68 ^h^	0.160	47.51

Mean of genotypes followed by the same letter within same row are not significantly different according to the least significant difference (LSD) (*P* < 0.05); CV, coefficient of variation; n.d.—not detected.

**Table 4:** Concentration of gliadin S-poor and S-rich subunits in grains of different bread and durum wheat genotypes (% of total extractable proteins).

	S-rich subunits (γ- + β- + α-gliadins)	S-poor subunits (ω-gliadins)	Sum (S-poor + S-rich)	S-poor/S-rich ratio
Bread wheat				
ZP 87/I	63.41 ^b^	0.60 ^d^	64.01 ^b^	0.017 ^c^
Apache	63.36 ^b^	0.50 ^d^	63.86 ^c^	0.008 ^d^
ZP *Zemunska rosa*	54.75 ^d^	0.83 ^c^	55.58 ^e^	0.015 ^c^
ZP 224	67.09 ^a^	2.53 ^a^	69.62 ^a^	0.038 ^a^
ZP Zlatna	59.11 ^c^	1.95 ^b^	61.06 ^d^	0.033 ^b^
*F*-test	***	***	***	***
CV (%)	7.24	57.29	7.77	54.29

Durum wheat				
ZP 34/I	60.78 ^a^	3.65^c^	64.43 ^a^	0.060 ^e^
ZP 10/I	52.56 ^d^	6.85 ^a^	59.41 ^d^	0.130 ^b^
ZP DSP/01	55.33 ^d^	6.99 ^a^	62.32 ^b^	0.126 ^a^
Varano	52.95 ^c^	5.70 ^b^	58.65 ^e^	0.108 ^c^
ZP 7858	54.98 ^b^	5.48 ^b^	60.46 ^c^	0.100 ^d^
*F*-test	***	***	***	**
CV (%)	5.23	39.96	6.12	26.48

Mean (bread wheat)	61.54 ^a^	1.28 ^b^	62.83 ^a^	0.022 ^b^
Mean (durum wheat)	55.32 ^b^	5.73 ^a^	61.05 ^a^	0.105 ^a^

Mean of genotypes and species followed by the same letter within same column are not significantly different (*P* < 0.05);

** = significant at *P* < 0.01;

*** Significant at *P* < 0.001; CV, coefficient of variation.

**Table 5:** Content of tryptophan and protein quality index in grains of different bread and durum wheat genotypes.

	Tryptophan (% d.w.)	QI (%)
Bread wheat		
ZP 87/I	0.138 ^d^	1.447 ^b^
Apache	0.150 ^bc^	1.621 ^a^
ZP *Zemunska rosa*	0.148 ^c^	1.171 ^c^
ZP 224	0.159 ^a^	1.352 ^b^
ZP Zlatna	0.141 ^cd^	1.154 ^c^
*F*-test	**	***
CV (%)	6.89	14.44

Durum wheat		
ZP 34/I	0.154 ^d^	1.268 ^b^
ZP 10/I	0.138 ^e^	1.245 ^b^
ZP DSP/01	0.172 ^b^	1.553 ^a^
Varano	0.163 ^c^	1.319 ^b^
ZP 7858	0.186 ^a^	1.496 ^a^
*F*-test	***	**
CV (%)	10.66	9.94

Mean (bread wheat)	0.147 ^b^	1.349 ^a^
Mean (durum wheat)	0.163 ^a^	1.376 ^a^

Mean of genotypes and species followed by the same letter within same column are not significantly different (*P* < 0.05);

** = significant at *P* < 0.01;

*** Significant at *P* < 0.001; CV, coefficient of variation.
